# A CBCT Investigation of the Association between Sella-Turcica Bridging and Maxillary Palatal Canine Impaction

**DOI:** 10.1155/2018/4329050

**Published:** 2018-02-22

**Authors:** Pamela M. Ortiz, Sawsan Tabbaa, Carlos Flores-Mir, Thikriat Al-Jewair

**Affiliations:** ^1^Private Practice, 2021 NW 136th Ave, Apt 597, Sunrise, FL 33323, USA; ^2^School of Orthodontics, Brooks Rehabilitation College of Health Sciences, Jacksonville University, Jacksonville, FL, USA; ^3^Division of Orthodontics, School of Dentistry, University of Alberta, Edmonton, AB, Canada; ^4^Department of Orthodontics, School of Dental Medicine, State University of New York at Buffalo, Buffalo, NY, USA

## Abstract

**Objectives:**

To investigate the association between unilateral/bilateral maxillary canine impaction and sella-turcica bridging using CBCT imaging.

**Methods:**

This retrospective comparative study analyzed 76 CBCT images of the craniofacial complex including sella-turcica. The impacted cuspid group consisted of thirty-eight subjects (7 males, 31 females; mean age, 14.6 ± 3.2 years) diagnosed with unilateral (left *n* = 14, right *n* = 11) or bilateral (*n* = 13) palatal canine impaction. The control group included thirty-eight subjects matched by sex (7 males, 31 females; mean age, 19.5 ± 3.6 years) with no impaction. Multinomial logistic regression analysis was used to determine the association between unilateral/bilateral canine impaction and right and left sella-turcica bridging.

**Results:**

The prevalence of sella-turcica bridging was 59.3% and 50% in the impacted canine and control groups, respectively. Although the odds for unilateral canine impaction were increased in the right and left sella-turcica bridging groups compared to the controls, the difference was not statistically significant. The risk of bilateral impaction was different between the two sides of sella-turcica bridging, but, again, the findings were not statistically significant.

**Conclusion:**

Contrary to previous 2D studies, there is no statistically significant association between unilateral/bilateral palatal canine impaction and sella-turcica bridging when using 3D CBCT.

## 1. Introduction

Impaction of maxillary canines is an anomaly that affects approximately 1-2% of the general population [1]. If left untreated, canine impaction may facilitate tooth malpositioning, root resorption, tooth mobility, arch-length discrepancy, and dentigerous cyst formation [2].

The sella-turcica is a saddle shaped depression centrally located within the sphenoid bone. The variance in the developmental and morphological patterns of the sella-turcica has been well documented and classified. Axelsson et al. [3] reported five distinct morphological variations of the sella-turcica including sella-turcica bridging, oblique anterior wall, double contour of the floor, irregularity in the posterior part of the dorsum sella, and pyramidal shaped dorsum sellae. The sella-turcica bridging variant is the focus for this study.

The sella-turcica bridging refers to the ossification of the ligament between the anterior and posterior clinoid processes [4]. This anomaly, in an otherwise healthy population, occurs with a reported incidence that ranges from 1.1 to 13% [3, 5–7]. However, the sella-turcica bridging has been associated with other systemic conditions, craniofacial aberrations, and, more specifically, dental anomalies such as palatal canine impaction [8–13].

Several theories describe the possible link between sella-turcica and palatal canine impaction. Neural crest cells are involved in the development of both the anterior wall of the sella-turcica and development of dental progenitor cells which form the teeth, therefore sharing a common embryologic origin [14]. Also, certain genomic mutations in the homeobox gene expression, contained within neural crest cells, can lead to faulty signaling pathways disrupting the development of midface, teeth, and sella-turcica [15, 16].

Previous radiographic studies [8, 9, 17] have investigated the possible association between sella-turcica bridging and dental anomalies including palatal canine impaction and have reported an increased frequency of bridging in subjects with dental anomalies. The radiographic studies thus far have all used conventional 2D lateral cephalograms as the means of determining the bridging. Although cephalometry is a routine diagnostic tool in orthodontics [18, 19], its utility to diagnose sella-turcica bridging is limited due to projection and magnification errors and potential overlap of structures [9, 10], and due to their 2D nature, data on right versus left bridging can not be obtained. Recent advances in craniofacial imaging have made it possible to obtain 3D representations of craniofacial structures with cone beam computed tomography (CBCT) and address the limitations of 2D lateral cephalometry.

Therefore the aim of this retrospective cross-sectional study was to assess the occurrence of sella-turcica bridging using 3D imaging in patients with maxillary canine impaction.

## 2. Materials and Methods

### 2.1. Sample Selection

This retrospective study was approved by SUNY at Buffalo Institutional Review Board. The CBCT images from two groups of subjects were compared. The impacted canine group included 38 active orthodontic patients diagnosed with maxillary palatal canine impaction (7 males and 31 females; mean age 14.6 ± 3.2 years; range 10.4–29.9 years). The control group consisted of 38 subjects without canine impaction (7 males and 31 females; mean age 19.5 ± 3.6 years; range 14.0–27.7 years).

The inclusion criteria for the treatment group were patients between 10 and 30 years of age selected from one private oral and maxillofacial surgery office with available CBCT images including the sella-turcica and the maxillomandibular region and a final diagnosis of unilateral or bilateral palatal canine impaction. Palatal canine impaction was defined as crown located palatal to adjacent teeth, more than 3/4 or complete root development, and the angle between the long-axis of the canine and the midsagittal plane (*α*-angle) > 45 degrees, obtained from simulated panoramic images reconstructed from CBCT data.

Subjects were excluded if they had severe craniofacial anomalies [13], history of orthognathic surgery, evidence of cleft lip and palate, maxillofacial pathology, trauma, missing teeth other than third molars, supernumerary teeth, and scans displaying motion artifacts in the areas of interest.

For the control group the same exclusion criteria applied and furthermore excluded any subject who lacked a full permanent dentition (except wisdom teeth) or exhibited any indications of previous orthodontic treatment. The scans of subjects in this group were solely taken for the purpose of third-molar extraction.

A total of 218 patients met the age criterion. After applying our inclusion/exclusion criteria, 38 subjects were included in the impacted canine group and diagnosed with unilateral (left *n* = 14, right *n* = 11) or bilateral (*n* = 13) palatal canine impaction. The control group consisted of 38 subjects chosen at random from a sample pool of 218 patients and matched to the treatment group by sex.

### 2.2. CBCT Imaging Acquisition and Evaluation

The retrospective CBCT images were acquired by an experienced technician using an i-CAT cone beam 3D Dental Imaging System version 3.1.62 (Imaging Sciences International, Hatfield, PA, USA). The i-CAT unit operated at 120 kVp, 3–7 mA, and a focal spot of 0.5 mm. All scans were taken at 0.4 mm voxel size for 40 seconds, with the exception of three scans (treatment group: 2; control: 1) that were taken at 0.3 mm voxel size for 40 seconds to acquire the raw data. The field of view was a cylinder 13 cm high and 16 cm in diameter. The grey scale range of the acquired image was 14 bits.

All scans were taken with the patients seated in an upright position with their heads oriented so that the occlusal plane was parallel to the floor, having the Frankfort horizontal plane slightly tilted. All scans were taken with the teeth out of occlusion by having the patient bite down on a cotton roll.

Each scanned image was exported to a Digital Imaging and Communications of Medicine (DICOM) file and then uploaded onto a computer running a Windows 7 (Microsoft, Redmond, WA, USA) operating system with a Dell LCD monitor (model U2713), with 2560 × 1440 resolution (Dell Computer Corp., Round Rock, TX, USA). The images were then imported into Dolphin 3D Imaging System version 11.7.05.66 Premium (Dolphin, Chatsworth, CA, USA). With this software, a fully reconstructed 3D image with sagittal, coronal, and axial slices was generated.

### 2.3. Landmark Identification and Examination of Sella-Turcica

The data pertaining to the maxilla, mandible, and their corresponding dentition were removed from the treatment and control scans. This allowed the investigator to be blinded to group assignment. The examiner waited a period of three weeks after the data were altered before evaluating sella-turcica.

To standardize the volume orientations, the axial plane (*x*) was set to the Frankfort horizontal plane. The midsagittal plane (*y*-axis) was set at the midpoint of sella-turcica; the midpoint was determined with a digital caliper. Yaw and roll were adjusted until the orbits were no longer overlapping (Figure 2).

**Figure 1:**
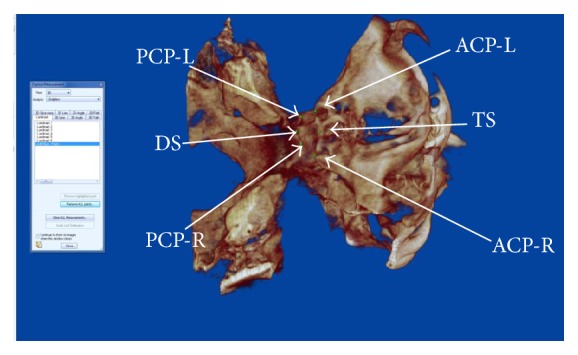
*Sella-turcica landmarks on CBCT*.* Note.* Tuberculum Sella Midsagittal (TS-MS) plane: midpoint on the anterior boundary of sella-turcica identified on the midsagittal plane; Dorsum Sella Midsagittal (DS-MS) plane: midpoint on the posterior boundary of the sell-turcica on the midsagittal plane; anterior clinoid process, right (ACP-R): the apex of the anterior clinoid process on the right side; anterior clinoid process, left (ACP-L): the apex of the anterior clinoid process on the left side; posterior clinoid process, right (PCP-R): the apex of the posterior clinoid process on the right side; posterior clinoid process, left (PCP-L): the apex of the posterior clinoid process on the left side.

**Figure 2:**
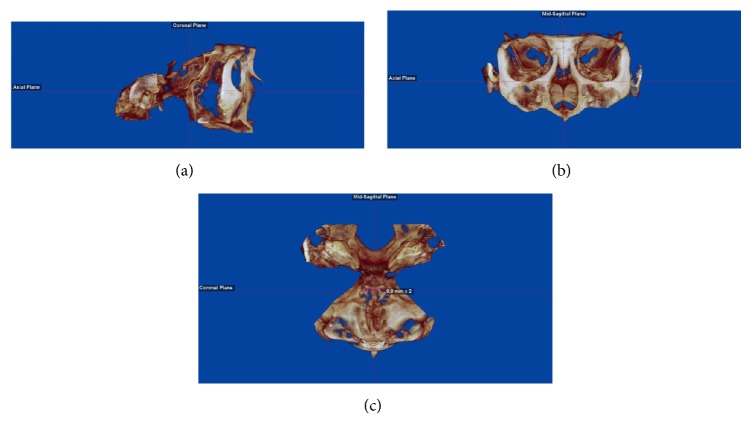
*Volume orientation*: (a) axial plane set to Frankfort horizontal plane (parallel to the floor using right sagittal view); (b) frontal view of axial and midsagittal plane; (c) midsagittal plane set using a digital caliper at the midpoint of sella-turcica.

Sella-turcica bridging was inspected via the 3D volumetric view. The three multiplanar views (sagittal, coronal, and axial) were configured to be displayed as 0.4 mm thick slices. Six landmarks (Figure 1 and Table 1) were identified in all views and marked with an onscreen 0.5 mm marker. The marker was checked for accurate anatomical placement by corroborating and adjusting its position in each multiplanar view. Once the landmarks were identified, their coordinate data were copied into an Excel spreadsheet. For each paired landmark the Euclidean distance *d* between the two points was calculated using Excel: $\left(d=\sqrt{{\left(x2-x1\right)}^{2}+{\left(y2-y1\right)}^{2}+{\left(z2-z1\right)}^{2}}\right)$. All data were measured by one examiner.

### 2.4. Sella-Turcica Bridging: Evaluation and Quantification

Sella-turcica bridging was classified into no bridging, partial bridging, and complete bridging. Due to the variation in size and shape of the clinoid processes and the inability to clearly discern where normal calcification ends and calcification pertaining to bridging begins, an objective-quantitative method for differentiating between the different bridging categories was developed.

The method quantified the calcification present in the right and left anterior and posterior clinoid processes individually and classified them into three groups using the ratio of interclinoid distance (ACP-PCP) to length (TS-DS). The ratios were complete bridging (ratio = 0%), partial bridging (ratio > 0 and <60%), and no bridging (ratio ≥ 60%). The cutoff point of 60% was chosen based on previous investigations [20].

### 2.5. Examiner Reliability

The interclass correlation coefficient (ICC) was performed to assess the intraexaminer reliability when measuring the distances between sella-turcica landmarks. One investigator remeasured twenty-one randomly selected scans from the impacted canine and control groups after a period of 4 weeks.

### 2.6. Statistical Analysis

Power calculations revealed that a sample of 34 subjects per group is needed to detect a difference of 31.6% between proportions at a power of 80% (5% significance level) [8].

Data were analyzed using SPSS software for Windows (version 23.0. Armonk, NY: IBM Corp). Data analysis suggested that data was normally distributed. The ANOVA test was used to compare mean left and right sella-turcica ratios between impacted canine/control and males/females within each group. Multinomial stepwise logistic regression analyses were used to determine associations between canine impaction and left and right sella-turcica bridging. The outcomes tested were no impaction, unilateral (right or left), and bilateral impaction. The bridging was dichotomized into no bridging and bridging (partial and complete combined) due to the small numbers of data in the complete bridging category. All tests were set at a significance of 0.05 with 95% CI.

## 3. Results

### 3.1. Intraexaminer Reliability

There was a high level of agreement for the repeated measurements between the first and the second time points (Table 2).

### 3.2. Descriptive Statistics

Table 3 describes the mean ages of the two comparison groups stratified by sex. The mean ages of females and males within the treatment and control groups were very similar. However, the ages of females and males were statistically significantly different when analyzed independently between the impacted and control groups (female control: 19.61 and female impacted canine: 14.48, *p* < 0.001; male control: 19.02 and male treatment: 15.2, *p* < 0.008).

### 3.3. Frequency of Sella-Turcica Bridging

Table 4 presents the overall frequency of sella-turcica bridging. Left sella-turcica bridging occurred in 57.9% (*N* = 44) of the subjects, of which 6.6% (*N* = 5) were complete bridging and 51.3% (*N* = 39) was partial bridging.  Table 5 presents the frequencies of each bridging category in the impacted canine and control groups. The frequency of complete sella-turcica bridging on the right side was higher for the control group, while it was similarly distributed between all groups on the left side (treatment and control).

The overall mean ratios for the impacted canine group were less than 60% and similar for both right (57.20%) and left (57.62%) sides, indicating partial bridging. The ANOVA test showed that the difference between the means (left ratio *p* = 0.256; right ratio *p* = 0.287) was not statistically significant. Mean ratios by group and sex are depicted in Table 6.

The overall mean left sella ratio for females was 59.43 compared to males 62.23. On the right sella, females displayed a mean ratio of 59.15 while males displayed a ratio of 62.52. The ANOVA test showed no statistically significant differences between females and males (left ratio *p* = 0.279; right ratio *p* = 0.258).

### 3.4. Association between Palatal Canine Impaction and Sella-Turcica Bridging

Table 7 shows the logistic regression models that tested the outcomes of bilateral and unilateral impaction for the predictors right and left sella-turcica bridging individually. The odds of bilateral canine impaction were 2.25 (95% CI [.59, 8.58]) for those with left sella bridging and .86 (95% CI [.24, 3.02]) for those with right sella bridging. Unilateral impaction was associated with higher odds of right and left sella bridging. However, none of the observed findings were statistically significant. Therefore the alternative hypothesis was rejected and there was no evidence of an increased risk of bridging on the right or left sides of sella when a canine impaction is present.

## 4. Discussion

Anatomical and cephalometric evidence of sella-turcica bridging has typically been reported as an anomalous finding, and its presence has been linked to various entities including syndromes, craniofacial, and dental abnormalities [9–11, 13, 21–24]. Some studies have advocated using the presence of sella-turcica bridging as a diagnostic marker to alert clinicians of the potential presence of other disease entities/anomalies [8].

This is the first study to assess the occurrence of sella-turcica bridging in orthodontic patients with palatally impacted canines using CBCT. This imaging modality allowed for an accurate assessment of sella bridging and addressing the problem of structure superimposition and thus false positive findings. The difficulty in discriminating between true bridging (fusion of the anterior and posterior clinoid processes) and pseudo bridging (superimposition of the interclinoid ligaments) on 2D cephalograms has been considered a limitation.

The logistic regression analyses of canine impaction (bilateral, unilateral, and no impaction) and the combined bridging categories showed higher occurrence of impaction in the presence of right and left sides of sella-turcica bridging when compared to controls and a lower occurrence of bilateral impaction in the presence of right sella bridging. However, none of these differences reached statistical significance. This finding is in disagreement with several 2D studies [8, 9, 17] that found a statistically significant increase in the incidence of sella-turcica bridging among subjects with palatal canine impactions and tooth transposition. Leonardi et al. [9], after evaluating lateral cephalograms of Caucasians, reported increased incidence of sella-turcica bridging in individuals with palatally displaced canines (complete: 5.6%; partial: 77.8%) compared to controls (complete: 9.9%; partial: 33.7%). Similarly, Ali et al.'s [8] study of Pakistani patients found a higher prevalence of sella-turcica bridging in cases diagnosed with palatal canine impaction (complete: 25.8%, partial: 54.8%) compared to (complete: 0%; partial: 51.4%) sella-turcica bridging in the control group. Najim and Al-Nakib [17] studied the same association in an Iraqi population and their findings concur with the previous finding. Increased prevalence of sella-turcica was observed in impaction cases (complete: 5%; partial: 65%) compared with controls (complete 0.8% and partial 27.5%).

To explain these results, the following has to be considered: variance in age and genetic make-up between our samples and the previous samples; differences in bridging measurement and classification methods such as combining partial and complete bridging groups in analysis of sella bridging association with impacted canines. Additionally, it is not stated in all the 2D radiographic studies that data collection was conducted blindly. 2D cephalometric radiographs do not have the advantage of eliminating data that might lead to the potential for bias. The use of CBCT in our study allowed us to digitally separate the dentition and maxillofacial structures from the sella-turcica scans. Thus, the examiner was blind to group assignments, which reduced the potential for bias. All these factors may have contributed to the heterogeneity between our study and the previous studies.

This study suggests that other factors warrant investigation when discussing the association and occurrence of maxillary canine impaction and sella-turcica bridging. Sharing common embryologic origins and gene mutations may not justify the link and the two findings maybe occurring independent from one another. The etiology of canine impactions is not fully understood, but there are a number of factors that were identified as possible etiologies, such as failure of root resorption in primary teeth, abnormal eruption path, presence of supernumerary teeth, crowding, oversized dental follicle, and genetics, in addition to other factors [1, 2]. Similarly, sella bridging can be the result of physiological activities of the chemical compounds that are involved in the embryogenesis and buildup of bone [25]. Future studies are needed to clarify the etiologies and further investigate the link.

The occurrence of complete sella-turcica bridging among the impacted canine group categories (unilateral left, unilateral right, and bilateral) and the control was different when comparing right and left sides of the sella-turcica. The left side frequency was equally distributed among all four groups. When combining all three impaction groups, the occurrence with complete sella bridging was 7.9% compared to 5.3% in the controls. For the right side, there was more occurrence of complete bridging in the controls (*N* = 5, incidence = 13.2%) when compared to all three impaction groups individually (unilateral left *N* = 0, unilateral right *N* = 0, and bilateral *N* = 1) and combined (incidence = 2.64%). This combined incidence was lower than the incidences reported by Ali et al. (25.8%, 0%), Najim and Al-Nakib (5%, 0.8%), and Leonardi et al. (16.7%, 9.9%) for treatment and control groups, respectively [8, 9, 17]. Sella-turcica bridging can occur in different forms and thus a bridge of the middle clinoid processes may have been misclassified in the previous 2D studies as a bridge of the anterior and posterior clinoid processes, thus inflating the results. It has to be stated however that these studies used patient-level evaluations while the current study used site-level statistics (right sella, left sella); thus direct comparison between findings may not be possible.

The overall frequency of partial bridging in our control group was 40.6%. Cederberg et al.'s [6] analysis of 225 randomly selected lateral cephalograms of pretreatment orthodontic patients reported an incidence of 68.8%. However, when compared to anatomical studies, our results were higher than the reported findings [26, 27]. When looking at the palatally impacted canines group, the partial bridging frequency in this study was 54%, which is comparable to Ali et al. (54.8%) but lower than two other studies that reported findings of 77.8%% and 70%, respectively [8, 9, 17].

Until present, there is no standardized objective system for classifying sella-turcica bridging. Lang [26] described the presence of a “central suture” or fissure centrally located in complete sella-turcica bridges, a feature which may or may not be present. Meanwhile, Ossenberg defined “complete” bridges as “completely fused boney projections” and “incomplete” bridges as those containing the suture or fissure described by Lang. Incomplete bridging has also been defined by other investigators as boney projections extending toward the opposing clinoid processes with a space in between [28]. These ambiguities in categorization and nomenclature make an accurate comparison between our study and previous studies difficult.

CBCT allowed for viewing the sella-turcica in three dimensions and free of superimpositions, making the diagnosis of complete bridging easy. However, distinguishing between a partially bridged sella and a nonbridged one proved to be difficult. Archana et al. [28] and Kolagi et al. [25] in autopsy studies of human dry skulls used visual methods for sella-turcica bridging categorization. This poses a great challenge in discriminating between a partial bridge and no bridge and thus may introduce measurement errors and bias. Also, not only do the clinoid processes appear to have great variation in shape and morphology, but also it is commonly unclear where a clinoid process ends and where the bridging begins [26].

Our study developed an objective-quantitative method to categorize sella-turcica bridging that accounted for the interclinoid calcification and used a 60% cutoff to differentiate between partial and no bridging based on the findings of Camp [20] who reported a normative mean interclinoid distance of 6.6 mm and sella length of 10.7 mm.

Variability in the incidence of sella-turcica bridging among different populations has been reported in the literature [25, 29]. A study on a Japanese population (male 3.9%, female 6%) has shown very low incidences of bridging while the same study found an Ontario Iroquois population (male 34.9%, female 31.7%) to have a relatively higher incidence. Further, due to their higher radiation dose when compared to conventional radiography, CBCT scans displaying the entire craniofacial region are rare. Even when they are indicated [30], most do not include sella-turcica, and when they do they are mostly of patients with a craniofacial abnormality or syndrome. Therefore, matching the samples by both age and ethnic background was not attempted. Future studies with larger and more homogenous comparison groups (e.g., homogenous ethnic groups) are warranted to confirm or refute these associations.

## 5. Conclusions

Sella-turcica bridging occurred in 59.3% and 50% in the impacted canine and control groups, respectively. Despite higher odds of unilateral and bilateral canine impaction in the presence of sella-turcica bridging when compared to controls, none of the findings were statistically significant. Therefore it is suggested that there is no statistically significant association between maxillary palatal canine impaction and sella-turcica bridging.

## Figures and Tables

**Table 1:** Measured distances between sella-turcica landmarks.

Distances	Definition	Landmark
TS-MS to DS-MS	Length of sella-turcica	Tuberculum Sella Midsagittal (TS-MS) plane: midpoint on the anterior boundary of sella-turcica identified on the midsagittal plane; Dorsum Sella Midsagittal (DS-MS) plane: midpoint on the posterior boundary of the sell-turcica on the midsagittal plane

ACP-R to PCP-R	Interclinoid distance right	Anterior clinoid process, right (ACP-R): the apex of the anterior clinoid process on the right side; posterior clinoid process, right (PCP-R): the apex of the posterior clinoid process on the right side

ACP-L to PCP-L	Interclinoid distance left	Anterior clinoid process, left (ACP-L): the apex of the anterior clinoid process on the left side; posterior clinoid process, left (PCP-L): the apex of the posterior clinoid process on the left side

Complete sella-turcica bridging	Distance between ACP and PCP equals zero	

**Table 2:** Intraexaminer reliability (*N* = 21).

Measurement	ICC^*∗*^	95% confidence interval
Length (TS-DS-MS)	.957	(.896, .982)
Interclinoid right (ACP-PCP-R)	.995	(.988, .998)
Interclinoid left (ACP-PCP-L)	.991	(.977, .996)

^*∗*^ICC = intraclass correlation coefficient.

**Table 3:** Mean ages of control and treatment groups stratified by sex.

Sex	Group	*N*	Mean age (yrs)	Std. deviation	Min	Max
Female	Control	31	19.6	3.6	14.0	27.7
	Treatment	31	14.5	3.4	10.4	29.9
Male	Control	7	19.0	2.0	17.2	21.9
	Treatment	7	15.2	2.4	12.1	19.7

**Table 4:** Overall frequency of sella-turcica bridging based on 60% cut-off^*∗*^.

Sella-turcica bridging	Left sella	Right sella
	*N* (%)	*N* (%)
Complete bridge (ratio = 0%)	5 (6.6)	6 (7.9)
Partial bridge (ratio > 0 and <60%)	39 (51.3)	33 (43.4)
No bridge (ratio ≥ 60%)	32 (42.1)	37 (48.7)

Total	76 (100)	76 (100)

^**∗**^The cutoff point of 60% was chosen based on ratio of reported means 6.6 mm/10.70 mm (left and right interclinoid distance/length, resp.). The ratios are complete bridging (ratio = 0 mm), partial bridging (ratio greater than 0 but less than 60%), and no bridging (ratio at least 60%).

**Table 5:** Frequency of sella-turcica bridging by maxillary impacted canine location.

Sella-turcica category	Sella-turcica side	Maxillary palatal impacted canine									
		None		Left		Right		Both		Total	
		*N*	(%)	*N*	(%)	*N*	(%)	*N*	(%)	*N*	(%)
Complete bridge (ratio = 0%)	Left	2	(40)	1	(20)	1	(20.0)	1	(20)	5	(100)
	Right	5	(83.3)	0	(0)	0	(0)	1	(16.7)	6	(100)
Partial bridge (ratio > 0 and <60%)	Left	17	(43.6)	8	(20.5)	6	(15.4)	8	(20.5)	39	(100)
	Right	14	(42.4)	8	(24.2)	6	(18.2)	5	(15.2)	33	(100)
No bridge (ratio ≥ 60%)	Left	19	(59.4)	5	(15.6)	4	(12.5)	4	(12.5)	32	(100)
	Right	19	(51.4)	6	(16.2)	5	(13.5)	7	(18.9)	37	(100)

Total	Left	38	(50)	14	(18.4)	11	(14.5)	13	(17.1)	76	(100)
	Right	38	(50)	14	(18.4)	11	(14.5)	13	(17.1)	76	(100)

**Table 6:** Mean sella-turcica bridging ratios in the treatment and control groups.

Sella-turcica bridging	Sex	Group	Mean ratio (%)	Std. deviation	Std. error	95% confidence interval		Min	Max
						Lower	Upper		
Left	Female	Control	64.00	16.59	3.08	57.69	70.31	28.21	95.20
		Treatment	55.02	15.42	2.81	49.26	60.78	16.29	84.97
	Male	Control	54.37	14.53	5.49	40.94	67.81	28.74	68.25
		Treatment	73.22	16.99	7.60	52.13	94.32	53.50	94.40
		Total	59.91	16.62	1.97	55.97	63.84	16.29	95.20

Right	Female	Control	64.58	21.61	4.16	56.04	73.13	25.25	106.67
		Treatment	54.41	18.17	3.26	47.75	61.08	18.03	87.71
	Male	Control	53.51	21.40	8.74	31.05	75.97	25.54	75.90
		Treatment	71.57	25.98	10.61	44.31	98.84	20.71	91.82
		Total	59.73	20.95	2.50	54.73	64.73	18.03	106.67

**Table 7:** Multinomial stepwise logistic regression for odds of bilateral/unilateral maxillary canine impaction with left and right sella-turcica bridging.

Model	Independent variable	No impaction = reference (*N* = 38)							
		Bilateral				Unilateral (right or left)			
		*B*	exp(*B*)	95% CI	Sig.	*B*	exp(*B*)	95% CI	Sig.^*∗*^
1	Left sella bridging (ratio ≤ 60%)	.81	2.25	(.59, 8.58)	.235	.528	1.78	(.63, 5.01)	.276
2	Right sella bridging (ratio ≤ 60%)	−.15	.86	(.24, 3.02)	.811	.241	1.27	(.46, 3.51)	.641

^*∗*^Significance set at *P* < 0.05.
